# Limonin Enhances the Antifungal Activity of Eugenol Nanoemulsion against *Penicillium Italicum* In Vitro and In Vivo Tests

**DOI:** 10.3390/microorganisms9050969

**Published:** 2021-04-30

**Authors:** Yi Li, Runan Zhao, Yan Li, Zhiqin Zhou

**Affiliations:** 1 College of Horticulture and Landscape Architecture, Southwest University, Chongqing 400716, China; liyi1991@email.swu.edu.cn; 2 Key Laboratory of Horticulture Science for Southern Mountainous Regions, Ministry of Education, Chongqing 400715, China; 3 College of Food Science and Technology, Huazhong Agricultural University, Wuhan 430070, China; zrn951219@163.com (R.Z.); yanli@mail.hzau.edu.cn (Y.L.); 4 The Southwest Institute of Fruits Nutrition, Banan District, Chongqing 400054, China

**Keywords:** eugenol nanoemulsion, citrus blue mold, limonin, *Penicillium Italicum*, antifungal activity

## Abstract

*Penicillium italicum*, the cause of citrus blue mold, is a pathogenic fungus that seriously affects the postharvest quality of citrus fruit and causes serious economic loss. In this study, a eugenol nanoemulsion containing limonin, an antimicrobial component from citrus seeds, was prepared using a high-pressure microfluidizer and the antifungal activity of the nanoemulsions against *P. italicum* was evaluated based on the conidial germination rate, mycelial growth, and scanning electron microscopy analysis. The results showed that the minimum inhibitory concentration and the inhibition rate of limonin-loaded eugenol nanoemulsion was 160 μg/mL and 59.21%, respectively, which was more potent than that of the limonin-free eugenol emulsion. After treatment with the nanoemulsions, the integrity of the *P. italicum* cell membrane was disrupted, the cell morphology was abnormal, and the leakage of nucleic acid and protein was observed. In addition, the challenge test on citrus fruits revealed that the limonin-loaded eugenol emulsion inhibited citrus infection for longer periods, with an infection rate of 29.2% after 5 days. The current research shows that nanoemulsions containing limonin and eugenol have effective antifungal activity against *P. italicum*, and may be used as a substitute for inhibiting blue mold in citrus fruits.

## 1. Introduction

Blue mold caused by *Penicillium Italicum* (*P. italicum*) leads to a tremendous postharvest loss in citrus fruits [1,2]. A common means for disease control is the use of chemical synthetic fungicides, such as imazalil, thiabendazole, pyrimethanil, and fludioxonil [3]. However, chemical fungicides may pose the threat to human health and the environment and cause fungal resistance as they are usually designed for a specific target of the pathogen [4,5,6]. At present, a few *Penicillium* strains have been proved to be resistant to chemical fungicides [7,8,9]. Given this, natural phytochemicals with antifungal activity have been taken into consideration, such as plant essential oils, phenolics, flavonoids, alkaloids, and terpenoids, etc. [10,11,12,13,14].

Eugenol (4-allyl-2-methoxyphenol), a naturally occurring phenolic compound that mainly exists in clove essential oil, has been reported to have excellent antifungal activity against strains of *Botrytis*, *Trichophyton*, *Penicillium*, *Aspergillus*, and *Fusarium* [15,16,17]. Due to the limitations of poor water solubility and stability as well as the high volatility of eugenol, current research mainly focused on improving its characteristics and further expand its applications using nanotechnologies [18,19]. A promising way is nanoemulsions, which enable the solubilization of eugenol in water by forming nanoscale oil droplets dispersed in the surfactants-containing aqueous phase. Limonin (7,16-Dioxo-7,16-dideoxylimondiol), a type of triterpenoid derived from the waste of citrus processing, such as seeds and peels, exhibits anti-inflammatory [20], antiviral [21], anticancer [22], and antimicrobial activity [23]. The underlying mechanism of its antifungal activity involves the breakage of energy production-related enzymes, destruction of the integrity of biomembranes, and interference with structural compound synthesis [24]. Nevertheless, limonin is rarely used in practice because of its extreme water insolubility. In our previous experiments, we found that limonin was able to dissolve in eugenol at ambient temperature conditions.

Combined antimicrobial agents may improve the antimicrobial activity, reduce the risk of the emergence of drug-resistant strains as well the dose of fungicides needed [25,26]. The synergistic effect between EOs with other EOs at low dosages exhibited a strong inhibitory effect on microbes [27,28]. Moreover, the combination of volatile compounds and non-volatile antimicrobial components enhanced the antimicrobial efficacy for most pathogens and had the potential to maintain the long-term effectiveness of antimicrobial agents [27,29]. Considering that when the eugenol nanoemulsion was used to treat citrus fruits under standard storage conditions, the volatility of eugenol remains a barrier to its long-term antifungal activity, we hope to design novel antifungal agents against *P. italicum* by adding the non-volatile component limonin.

In this study, a eugenol nanoemulsion was prepared using a high-pressure microfluidizer. The antifungal activities of the eugenol nanoemulsion and limonin-loaded nanoemulsion were investigated in the substrate and infected citrus, respectively. Besides, the possible mechanisms involving morphological changes, intracellular constituent losses, and biomembrane permeability in *P. italicum* were also studied. The results may provide a reference for the development of safe natural fungicides with high efficiency.

## 2. Materials and Methods

### 2.1. Materials

Eugenol (EG, ≥99% GR assay) was supplied by Aladdin Reagent Database Co. (Shanghai, China). Limonin (LM, ≥97%) was purchased from Rundekang Biotechnology Co., Ltd. (Baoji, China). Paraffin oil (PA) and Tween 80 (TW80) were purchased from Kelon Chemical Reagent Factory (Chengdu, China). Potato dextrose agar (PDA) and potato dextrose broth (PDB) were from Bowei Biotechnology Co., Ltd. (Shanghai, China). Distilled water with a resistance of 18.2 MΩ-cm was purified using a Milli-Q water-purification system (Bedford, MA, USA) and used to prepare all aqueous solutions.

Nanfeng mandarin (*Citrus reticulata* Blanco) fruits at commercial maturity were harvested from a local market in Nanfeng City (Jiangxi, China) in early December 2019. Orange fruits free of mechanical injury and without any disease were selected based on the weight (50–60 g) and visible similarity in color. The fruits were then dipped in 1.0% (*v*/*v*) sodium hypochlorite solution for 2 min, washed with running tap water, and air-dried at 25 °C before the challenge test.

### 2.2. Nanoemulsion Preparation

Nanoemulsions were prepared according to a previously reported method with some modifications [30]. Briefly, 40 mg limonin was dissolved in 4 g eugenol, and mixed with 1 g paraffin oil, and stir well. Then 95 g TW80 aqueous solution (1%, *m*/*v*) was added to the mixture before homogenization with a high-speed disperser (T18 digital ULTRA TURRAX, IKA Instruments Ltd., Staufen, Germany) at 12,000 rpm for 3 min in an ice bath. The obtained coarse pre-emulsions were homogenized by passing through a high-pressure microfluidizer (Microfluidics M110L, Microfluidics Corp., Newton, MA, USA) at 9000 psi for five passes. EG nanoemulsions without limonin were also prepared in the same way. Subsequently, all nanoemulsions were stored at 4 °C for analysis. For convenience, EG and EGL nanoemulsions mentioned below were limonin-free and limonin-loaded nanoemulsions, respectively. Note that the concentration of limonin in the initially prepared EGL nanoemulsion was calculated to be 0.4 mg/mL, and the concentration of eugenol was 40 mg/mL. In subsequent experiments, the original emulsion was diluted using eugenol concentration as a reference.

### 2.3. Characterization of Nanoemulsions

#### 2.3.1. Particle Size Determination

The particle size of the nanoemulsion droplets was measured at 25 °C by dynamic light scattering at an angle of 173° using a Nano Zetasizer ZS (Malvern Instrument, Malvern, UK), including particle size and polydispersity index (PDI). Nanoemulsions were diluted 500 times with deionized water before measurement to avoid multiple scattering effects.

#### 2.3.2. Optical Microscopy and Confocal Laser Scanning Microscopy

Visualization of nanoemulsion droplets was carried out using an optical microscope (NIKON ECLIPSE 80i, magnification 40×) and a U-TB04917 confocal laser scanning microscope (CLSM, Olympus Corporation, Tokyo, Japan). For CLSM, the nanoemulsion was mixed with Nile red (0.01 wt% dissolved in alcohol) and thoroughly stained by gently shaking the mixture for 2 min. The excitation and emission wavelengths of Nile red were 543 nm and 578 nm, respectively. Images were taken at a magnification of 100× of the objective lens.

### 2.4. Antifungal Activity of the Nanoemulsions

#### 2.4.1. Fungal Strains

*P. italicum* was isolated and purified by the Key Laboratory of Horticulture Science, College of Horticulture and Landscape Architecture, Southwest University (Chongqing, China). Fungal strains were grown on PDA plates and stored at −20 °C. For experiments, the fungal spores were collected after incubation on PDA for 4 days at 28 °C and harvested in sterile distilled water containing 0.1% TW80.

#### 2.4.2. Determination of MIC and MFC

The antifungal activity against *P. italicum* was determined by a broth dilution assay in 96-well plates with some modifications [31]. Briefly, 200 μL of a mixture consisting of EG or EGL nanoemulsions and PDB were prepared in the first well of the column, making the concentration of eugenol 2560 µg/mL. Next, 100 μL PDB was added to the rest of the wells of the 96-well plate, and 100 μL mixed solution was transferred from the first well to the second one and so on. Then, 100 μL spore suspension (10^5^ CFU·mL^−1^) was added to each well and mixed thoroughly to obtain final concentrations of eugenol ranging from 80 µg/mL to 1280 µg/mL. Then, the 96-well plates were covered and incubated at 28 °C for 72 h. Experiments of each sample were carried out in triplicate. PDB mixed with the conidial suspension was used as the positive control, while the negative control included PDB only. The minimum inhibitory concentration (MIC) was defined as the lowest concentration showing no visual growth of *P. italicum*. Then, 100 µL of the mixture from the well with no visual growth of the fungus was inoculated onto a PDA plate and cultured for another 72 h. The minimum fungicidal concentration (MFC) was defined as the lowest concentration showing no fungal growth on the PDA plate.

#### 2.4.3. Influence of the Emulsions on Conidial Germination of *P. italicum*

The inhibitory effects of nanoemulsions on the spore germination of *P. italicum* were evaluated using the concavity slide method [32]. Briefly, a conidial suspension (10^7^ CFU·mL^−1^ in sterilized PDB medium) was freshly prepared, and 100 μL of the nanoemulsion and conidial suspension mixture was prepared in glass depression slides, making the final concentration of eugenol 160 µg/mL. Meanwhile, the EGL treatment group also contained 1.6 µg/mL of limonin. Conidial suspension with equal amounts of sterile water was used as the control. Then, the depression slides were placed in Petri dishes (9 cm in diameter) containing wet filter paper and cultured at 28 °C. Conidial germination was evaluated under an optical microscope (NIKON ECLIPSE 80i) at 5 h, 10 h, and 24 h. A conidium was considered to have germinated when the germ tube was at least as long as the length of the conidia.

#### 2.4.4. Inhibitory Effect of the Nanoemulsion on Mycelial Growth of *P. italicum*

The inhibitory effect of the nanoemulsion on the mycelial growth of *P. italicum* was determined according to a previous method [33] with some modifications. In brief, the sterilized and cooled PDA medium was added to several Petri dishes (5 cm in diameter). Then, a certain volume of EG or EGL nanoemulsion was mixed with PDA, making the final concentration of eugenol was 80 µg/mL and 160 µg/mL, respectively. Correspondently, 80 µg/mL and 160 µg/mL EGL groups also contained 0.8 µg/mL and 1.6 µg/mL of limonin, respectively. Plates with only PDA medium were set as controls. After medium solidification, a 7-day-old mycelial disk (5 mm in diameter) of *P. italicum* was placed on the medium in each Petri dish and all plates were incubated at 28 °C for one week. The colony diameters of each group were measured and recorded after 3 days of incubation. Three replicates were carried out for each treatment. The following formula was used to calculate the inhibition rate of mycelial growth:

(1) $$Inhibition\;rate\;of\;mycelial\;growth\;\left(\%\right)=\frac{Dc-Dt}{Dc-Di}\times 100$$

where *Dc* and *Dt* are the mean colony diameters of the control and treated groups, respectively. *Di* is the initial diameter of the mycelial disk.

### 2.5. Morphological Observation

A scanning electron microscope (SEM) was used to observe the changes in mycelia and conidial morphology of *P. italicum* [34]. For SEM, *P. italicum* was incubated on PDA treated with EG or EGL nanoemulsion (eugenol concentration in both at 160 µg/mL) at 28 °C for 5 days, and samples without nanoemulsion treatment were used as controls. Segments of ~2 mm^3^ were cut from fungal cultures grown on the PDA plate and fixed with a 2.5% glutaraldehyde solution overnight at 4 °C. Then, the samples were washed with phosphate-buffered saline (PBS, 0.1 M, pH 7.0) twice for 10 min each. Thereafter, the samples were fixed with 1% precooled osmic acid at 4 °C for 1 h, washed twice with PBS, and then passed through a graded series of alcohol for dehydration. Next, the tissues were dried and gold-covered by vacuum plating (10 KV, 220 s). The morphology of *P. italicum* was observed using a scanning electron microscope (Hitachi SU8020, Tokyo, Japan).

### 2.6. Determination of Microbial Cell Membrane Permeability

#### 2.6.1. Extracellular Electric Conductivity Measurement

The extracellular conductivity of *P. italicum* was determined according to a method described previously [1] with some modification, using a model DDS 309^+^ electric conductivity meter (Century Ark Technology Co. Ltd., Chengdu, China). After 48 h of shaking incubation at 28 °C in PDB, the mycelia were collected and washed three times with water, and the moisture on the surface was then removed with absorbent paper. Then, 0.4 g of the wet mycelia were re-suspended in 40 mL pure water, and a certain volume of nanoemulsions was added to make the final concentrations of eugenol 160 µg/mL. After 4, 8, 12, 24, 36, 48, and 60 h incubation in a shaker at 28 °C, 5 mL *P. italicum* mycelial suspensions were collected and centrifuged at 13,800× *g* for 10 min. The supernatant was used to determine the electrical conductivity. Subsequently, total electrical conductivity was measured after the mycelia were boiled for 10 min (for complete destruction) and cooled to 25 °C. The mycelia untreated with nanoemulsion were used as the control, and the extracellular electric conductivity was expressed as a percentage of the relative electric conductivity (REC), according to the following formula:

(2) $$Relative\;electric\;conductivity\;\left(\mathrm{REC},\%\right)=\frac{\left(R_{t}-R_{0}\right)}{\left(R_{k}-R_{0}\right)}\times 100$$

where *R*_0_ is the electric conductivity of pure water (25 °C), *R_t_* is the electric conductivity of the treatments at different culture times, and *R_k_* is the electric conductivity of the boiled mycelia.

#### 2.6.2. Leakage of Protein and Nucleic Acid

According to a modified method [35], the leakage assay was performed using spectrophotometry. Briefly, the mycelia were freshly prepared as mentioned above and washed three times with PBS (0.1 M, pH 7.0). Then, 0.4 g of the wet mycelia were re-suspended in 40 mL PBS (0.1 M, pH 7.0). Subsequently, EG and EGL nanoemulsions were added to make the final concentrations of eugenol at 160 µg/mL. Additionally, the interference of nanoemulsions to the absorption value was also investigated using EG or EGL nanoemulsions in PBS, respectively. After incubation for 0, 4, 8, 12, 24, 36, 48, and 60 h, the mycelial suspensions of different groups were centrifuged, and the supernatant was used to measure the absorbance at 260 nm and 280 nm. PBS was set as blanks. The data was calculated as the absorbance value of the test solution minus the background absorption caused by the nanoemulsions. Nucleic acid leakage was expressed as the value of OD_260_, and the protein leakage was expressed as OD_280_.

### 2.7. Lipid Peroxidation Assay

Malondialdehyde (MDA) is an end-product of lipid peroxidation, and its content was determined using the thiobarbituric acid method described by [36] with some modifications. For the assay, *P. italicum* was cultured on a cellophane layer in a Petri dish with EG or EGL nanoemulsion (160 µg/mL)-treated PDA and incubated for 5 days at 28 °C. Then, 0.2 g of fresh mycelia were ground with 4 mL 10% (wt%) trichloroacetic acid aqueous solution and centrifuged at 13,800× *g* for 10 min. The supernatant (1 mL) was mixed with 2 mL 0.6% (wt%) thiobarbituric acid solution prepared in 10% trichloroacetic acid aqueous solution and the mixture was boiled in a water bath for 15 min. Then, the samples were transferred to a cold-water bath to terminate the reaction before centrifuging at 13,800× *g* for 10 min for determination. The supernatant of each sample was measured at 532, 450, and 600 nm. The amount of MDA was expressed as nmol/g mycelia using the following formula (Yan et al., 2020):

(3) $$\mathrm{MDA}\;content\left(nmol/g\right)=\left[6.45\times \left(OD_{532}-OD_{600}\right)-0.56\times OD_{450}\right]\times k\times \frac{V_{t}}{m}\;$$

where *V_t_* (mL) is the volume of the total extract solution, *k* is the dilution factor, and *m* (g) is the mass of fresh mycelia.

### 2.8. Challenge Test of Citrus Fruits

The selected fruits were cut (4 mm in diameter, 2 mm in depth) with three cuts evenly made at the equatorial side per fruit and then divided into three groups: control, EG-treated, and EGL-treated groups. For the treatment, oranges (8 fruits per replicate, *n* = 3) were injected with 10 μL nanoemulsion (320 µg/mL of eugenol in EG treatment, 320 µg/mL eugenol and 3.2 µg/mL limonin in EGL treatment, respectively.) into each cut, and the control group was injected with an equal volume of sterile water. Then, 10 μL spore suspension of *P. italicum* (10^5^ CFU/mL) was inoculated into the cut (Figure S2), and the fruits were then placed in a foam plastic box with 95% to 98% relative humidity at 25 °C. During the incubation period, the numbers of mildewed fruits and cuts with visible mycelium growth were recorded.

### 2.9. Statistical Analyses

All experiments in the present investigation were performed in triplicate and the data are shown as the mean ± standard deviation (SD). ANOVA was further performed on the present data using SPSS version 16.0 for Windows (SPSS Inc., IBM Corp., Armonk, NY, USA).

## 3. Results

### 3.1. Characterization of Nanoemulsions

**Figure 1:**
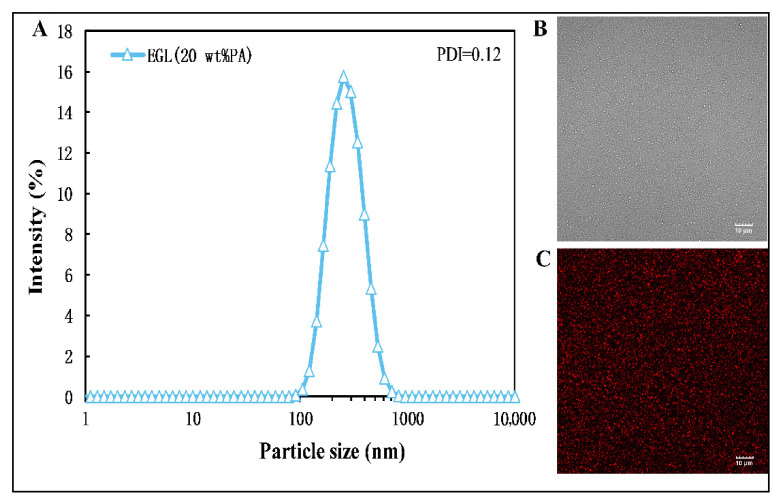
Particle size distribution (**A**) and microstructure (**B**,**C**) of limonin-loaded eugenol nanoemulsion. The mean particle size was 245.7 nm. Scale bar, 10 μm.

As shown in Figure 1A, the particle size distribution of the EGL nanoemulsion was typically monomodal with a narrow PDI (data = 0.12). The droplet size was 245.7 nm, markedly larger than that of the EG nanoemulsion without limonin (213.7 nm). In addition, the storage time (4 °C) had little influence on the microstructure of the EGL nanoemulsion according to uniform size droplets observed by CLSM microscopy after 14 days (Figure 1B,C).

### 3.2. Antifungal Activity

#### 3.2.1. MICs and MFCs Determination

In this study, the MICs and MFCs of nanoemulsions against *P. italicum* were determined to be within the range of 80 μg/mL to 1280 μg/mL (concentration of eugenol). After 3 days of incubation, mycelial growth was observed at 80 μg/mL and 160 μg/mL in the EG and 80 μg/mL in the EGL groups, respectively (Table 1). After being transferred to PDA and cultured for 3 days, mycelial growth was completely inhibited over 320 μg/mL of EG and EGL nanoemulsions. According to the definitions, both the MIC and MFC of EG nanoemulsion were 320 μg/mL, while the MIC of EGL emulsion was 160 μg/mL and the MFC was 320 μg/mL. This suggests that EGL nanoemulsion exhibited stronger activity than EG nanoemulsion.

#### 3.2.2. Inhibition of Spore Germination

**Figure 2:**
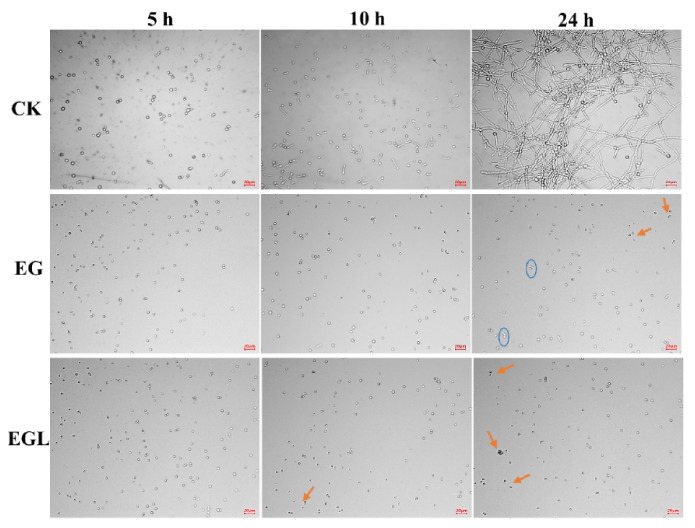
Optical microscopy of the spore germination after treatment with water (CK), eugenol nanoemulsion (EG), and limonin-loaded eugenol emulsion (EGL). Scale in the graph, 20 μm.

Using an optical microscope, conidial germination was examined after incubation for 5, 10, and 24 h of different treatments. As shown in Figure 2, 160 μg/mL of nanoemulsions significantly inhibited the spore germination of *P. italicum* in comparison to the control. The spore germination rates of CK at 5 h, 10 h, and 24 h were 8.32%, 52.09%, and 90.72%, respectively. However, the percentages of germinated spores in the EG group (blue circle indicated in the graph) were only 0, 5.63%, and 9.18%, respectively. Furthermore, the EGL nanoemulsion showed a more potent inhibitory activity than EG, as no spore germination was observed within 24 h. Additionally, more unhealthy spores (indicated by a small arrow) were present in EGL-treated groups, suggesting severe damage to spore structure.

#### 3.2.3. Inhibition of Mycelial Growth

**Figure 3:**
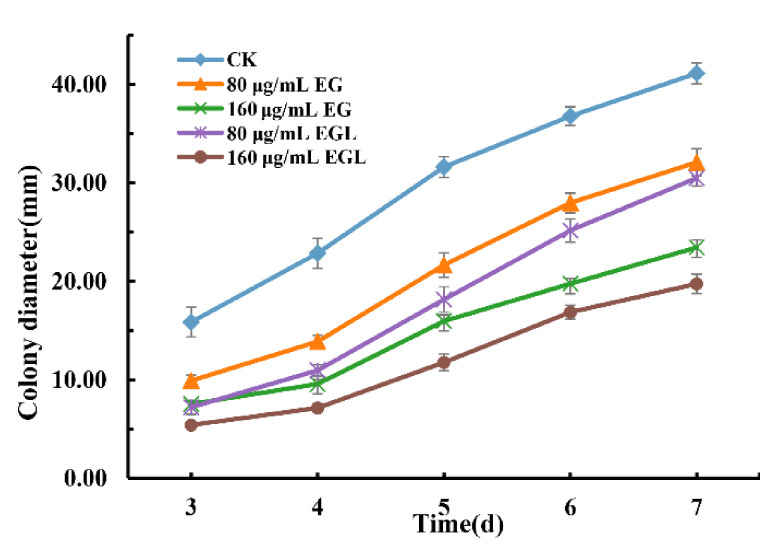
Colony diameters of *P. italium* after incubation for 3–7 d after different treatments.

The impact of the nanoemulsions on the mycelial growth of *P. italicum* is shown in Figure 3. The difference between the control and treated groups was significant (*p* < 0.05), showing that the mycelial growth of *P. italicum* was effectively inhibited by EG and EGL nanoemulsions. Meanwhile, groups treated with high concentrations (160 μg/mL) showed higher inhibition than those treated with a low concentration (80 μg/mL) in each nanoemulsion. Furthermore, EGL exhibited stronger antifungal activity than EG at the same level, and the colony morphology were obviously abnormal after treated with EG and EGL nanoemulsion (Figure S1). After incubation for 7 days, the inhibition rate of the nanoemulsion treatments calculated based on the colony diameter ranged from 25.02% to 59.21%.

### 3.3. Micromorphological Analysis by SEM

**Figure 4:**
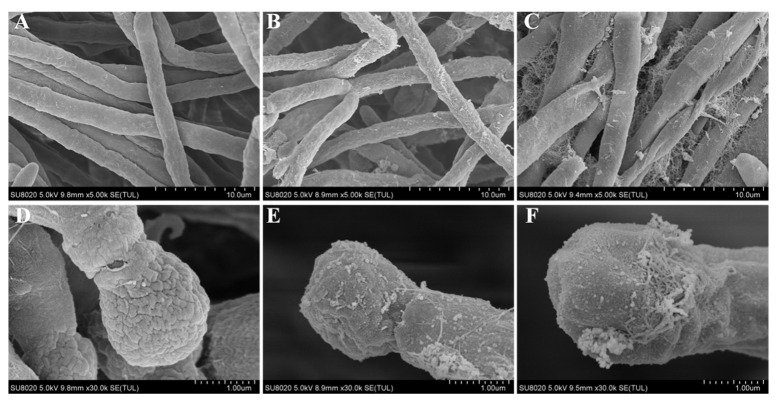
Morphology of the mycelia (**A**–**C**) and conidia (**D**–**F**) of P. italicum, as observed by scanning electron microscopy (SEM), after treatment with water (**A**,**D**), 160 μg/mL EG nanoemulsion (**B**,**E**), and 160 μg/mL EGL nanoemulsion (**C**,**F**). Scale bar, 10 μm for mycelia and 1 μm for spore.

The influence of EG and EGL nanoemulsions on the morphology of *P. italicum* was examined by SEM (Figure 4). The control fungus grown on PDA presented a normal morphology with smooth, tubular, and homogenous mycelia (Figure 4A) as well as mellow and sturdy conidia (Figure 4D). However, the mycelia and conidia of *P. italicum* treated with 160 µg/mL EG or EGL emulsion showed obvious morphological abnormalities. The surface of mycelia treated with EG was rough and unhealthy with white filaments attached (Figure 4B) and the conidium was atrophied (Figure 4E). In contrast, the degree of damage of *P. italicum* treated with EGL nanoemulsions was more severe with shriveled mycelia, plicated surface, presence of debris (Figure 4C), and shrunken conidium (Figure 4F). The result suggested that the combination of eugenol nanoemulsion and limonin destroyed the structure of *P. italicum*, which led to a massive loss of cellular contents.

### 3.4. Influence of Nanoemulsions on the Cell Membrane Permeability

#### 3.4.1. Extracellular Conductivity

**Figure 5:**
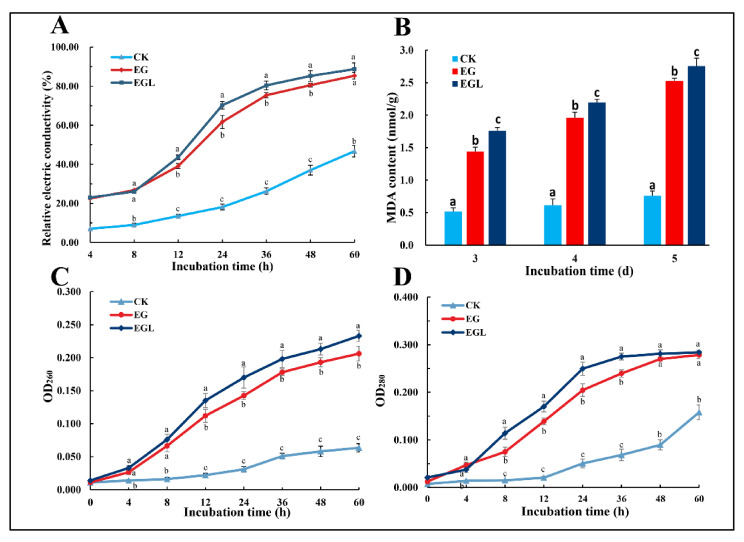
Influence of the nanoemulsion (at a eugenol concentration of 160 μg/mL) on the extracellular conductivity (**A**), lipid peroxidation (**B**), leakage of nucleic acid (**C**), and protein (**D**) of *P. italicum*. Data are shown as mean ± SD from three replications and different letters represent a significant difference (*p* < 0.05).

The extracellular conductivity of *P. italicum* treated by different methods was determined at 4, 8, 12, 24, 36, 48, and 60 h. As shown in Figure 5A, the REC of *P. italicum* gradually increased with increasing exposure time. After 8 h, the value after treatment with EG and EGL nanoemulsion quickly increased, which was significantly higher (*p* < 0.05) than that in the control. At 8 to 24 h, the increase rate (slope) of the REC in the EGL-treated group was higher than that in the EG-treated group. Although the increase rate of the latter showed a slight upward trend after 24 h, the REC value was still lower than that of the former. After 36 h, both increased at a slower rate than before. However, the REC of the control group remarkably increased in the late stage, which may be due to the programmed cell death of *P. italicum*. After 60 h of exposure, the REC values of EG-treated, EGL-treated, and the control were 87.0 ± 1.7%, 88.7 ± 3.2%, and 46.9 ± 2.9%, respectively.

#### 3.4.2. Leakage of Nucleic Acid and Protein

Leakage of the nucleic acid of *P. italicum* was evaluated by the OD260 (Figure 5C). After treatment with nanoemulsions, the absorbance value increased when the incubation time was increased from 0 to 60 h. The OD260 of the treated groups gradually increased at a higher rate than that in the control. After 60 h, the OD 260 of the EGL-treated group was 0.231 and 0.208 in the EG-treated group. The difference between the treated groups and the control was significant (*p* < 0.05). However, there was no significant difference between the EG-treated and EGL-treated groups within 8 h (*p* > 0.05). Thereafter, the leakage rate (the slope) of nucleic acids in the EGL-treated group was accelerated as the OD260 becoming higher than the EG group (*p* < 0.05).

The leakage of *P. italicum* protein was assessed by OD280 (Figure 5D). The absorption of the control increased slowly until 12 h, while the treated groups showed a significant increase in OD280 values after the nanoemulsion was added. The absorbance value of the EGL group rapidly increased from 4 h to 24 h and continued to rise until 36 h, after which the value remained stable. In contrast, the increase rate in the EG group was relatively low and the absorbance value was significantly lower than that in the EGL group from 8 h to 36 h. Owing to cell death, protein leakage quickly increased in the late period of the control (after 48 h).

### 3.5. Lipid Peroxidation

MDA content is known to be associated with oxidative stress in cells. After exposure to EG and EGL nanoemulsions, MDA content was significantly higher than that in the control (Figure 5B). The results indicated that lipid peroxidation that occurred in the treated group was more serious than that in the control group, and the degree of lipid oxidation further increased with an increase in the exposure time from 3 d to 5 d. P. italicum treated with EGL showed significantly higher MDA content than that treated with EG nanoemulsion (*p* values were 0.021, 0.014, and 0.040 in 3 d, 4 d, and 5 d, respectively).

### 3.6. Challenge Test

**Figure 6:**
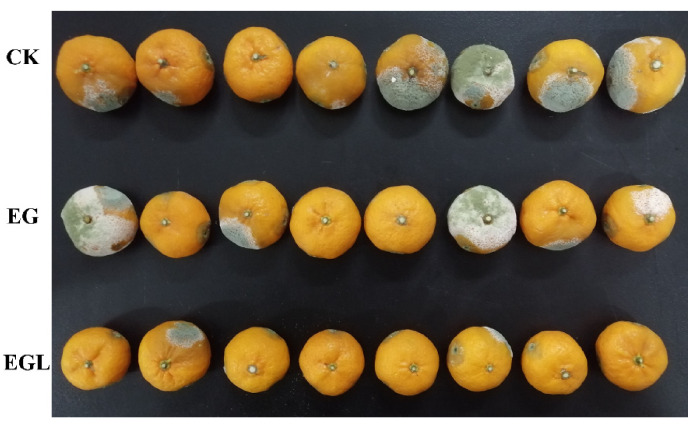
Challenge test of the citrus fruits treated with *P. italicum* after 5 d. Each group (8 fruits per replicate, *n* = 3) was injected with 10 μL nanoemulsion (320 µg/mL) into each cut, and the control group was treated with sterile water.

The percentages of infected citrus and infected cuts inoculated with *P. italicum* were measured after storage for 3 days and 5 days, respectively (Table 2). The mean morbidity of the control group was 75% after 3 days, while the whole control group was mildewed after 5 days. However, fruits treated with EG or EGL showed lower morbidity than the control fruits (*p* < 0.05). After 3 days, the mean percentage of mildew citrus treated with EG was 37.5%, but this was not significant when compared with the EGL-treated groups (*p* > 0.05). After 5 days, the infection rate of EGL-treated citrus was significantly lower than that of EG-treated citrus (Figure 6). Additionally, the percentages of infected cuts in the control group were 59.7% after 3 days and 84.7% after 5 days, respectively, which were significantly higher than those of citrus fruits treated with nanoemulsions. Moreover, the infection rates of cuts between the EG and EGL groups were significantly different (*p* < 0.05), indicating that the EGL nanoemulsion exerted more potent antifungal effects.

## 4. Discussion

Limonin, a highly oxygenated triterpenoid dilactone, is the first isolated limonoid and is also considered an important active ingredient in citrus seeds [37]. It has been reported for various biological activities like antiviral, antifungal, antibacterial, anticancer and antimalarial, etc. [38,39,40]. However, to our knowledge, there have been no reports on the application of limonin in reducing postharvest blue mold of citrus fruit. In the current study, limonin and eugenol, another antifungal phenolic compound, were encapsulated in a nanoemulsion. The nanoemulsion showed a remarkable inhibition against *P. italicum* by affecting the mycelial growth, spore germination, mycelium morphology, membrane permeability, and membrane lipid peroxidation.

According to previous reports, essential oils such as citral, eugenol, carvacrol, and thymol are effective in preventing fungal spore germination and mycelial growth [11,41], which are in agreement with our findings. And it is encouraging to compare the present results with that found by Moussa et al. who found that the percentage of mycelial growth inhibition of the free eugenol against *P. italicum* was 19.3% at 0.25 mg/mL and 10.6% at 0.125 mg/mL [10], which are lower than those measured in the present study. In general, EG nanoemulsions have better solubility and lower volatility than the single eugenol, making nanoemulsions more effective in exhibiting antimicrobial activity [42]. In addition, when citrus fruits are infected with *P. italicum*, blue mold disease occurs quickly and spreads under high humidity and optimum temperature [43]. Under the current experimental conditions, all control groups exhibited a blue mold after 5 days. By contrast, in the early stage, the EG nanoemulsion and the EGL nanoemulsion treatments delayed the occurrence of the disease. Furthermore, the inhibitory effect on the disease of the EGL was more prominent after incubation for 5 days. A reasonable explanation was that the EG nanoemulsion inhibited the growth of the fungus and affected the biofilm to a small extent, while the EGL emulsion severely destroyed the structure of *P. italicum* and made it impossible to regenerate, which was consistent with the results of scanning electron microscopy (Figure 4).

Essential oils have been reported to exert their antifungal activity against fungi based upon the influence on the cell membrane integrity and permeability [44]. This infiltration results in several intercellular negative consequences, like overall leakage of intracellular proteins, nucleic acids, ions, or other intercellular contents [11,45,46]. Our results showed that after treatment with nanoemulsions, the extracellular conductivity in *P. italicum* supernatants quickly increased. Additionally, the interference and destruction of the cell membrane by EGL were greater than that by EG nanoemulsion after 8 h. For the EG- and EGL-treated fungus, the leakage rates of both nucleic acid and protein increased after 4 h of exposure. This may be due to the accumulation of the active ingredient released from the nanoemulsion [18]. These findings suggested that the cell membranes of *P. italicum* were highly damaged and nucleic acid and protein leakage occurred after exposure to nanoemulsions [44]. Furthermore, with the action of nanoemulsions on the biofilm of *P. italicum*, the degree of lipid peroxidation in the cell membrane increased, leading to an increase in MDA content. This also accords with the previous study by Yan et al. [47], which showed that cell membranes of microorganisms undergo lipid peroxidation and structural destruction owing to the action of antimicrobial components. In addition, proper fluidity is necessary for the normal functioning of the biological membranes and can be regulated by fatty acids and sterols of the fungi [48]. Eugenol has been reported to inhibit the biosynthesis of ergosterol [49], a membrane sterol that is specific to fungi and being responsible for the regulation of membrane fluidity. Further studies are thus needed to elucidate the relationship between the nanoemulsions treatment and *P. italicum* membrane fluidity, and it is necessary to determine the ergosterol content in the following research.

According to the previous studies [27,43], combined antifungal agents may have two advantages: (i) They can reduce the dosage of agents needed and probably achieve a better inhibitory effect; (ii) They can reduce the risk of fungi resistance development by increasing the target and mode of action of antifungal agents. Our preliminary analyses with 20 μg/mL of limonin alone (LM are prone to be precipitated in the medium above this concentration) affected neither the growth of *P. italicum* on the PDA plate nor in the PDB medium. Nevertheless, the concentration of limonin in 320 μg/mL EGL nanoemulsion was 3.2 μg/mL, much lower than 20 μg/mL. Our results showed that the addition of a low dose of limonin to EG nanoemulsion could significantly improve the antifungal activity against *P. italicum* in vitro, suggesting that limonin and eugenol played a synergistic effect. Limonin has been reported to interfere with the transmission of signals between microbial cells and effectively inhibit intercellular communication [39]. Therefore, limonin probably reduced the adaptability of fungal cells to external pressures and enhance their sensitivity to eugenol. Besides, according to in vivo test, we found that the addition of limonin to essential oils can significantly increase its long-term effectiveness in the postharvest preservation of citrus fruits. For practical use, postharvest fruits may be treated by more conventional means, such as spraying or soaking with nanoemulsion. Despite these promising results, there are still many unanswered questions about the in-depth antifungal mechanisms against the *P. italicum*. In particular, according to our previous observation (Figure S1), EGL plays an important role in the growth and reproduction of *P. italicum*, a further study with more focus on the metabolites of the fungi may be carried out.

## 5. Conclusions

In conclusion, our results indicate that limonin-loaded eugenol nanoemulsions exhibited excellent antifungal activity against *P. italicum* and effectively inhibited the occurrence of citrus blue mold infection caused by *P. italicum* spores. The antifungal mechanism was related to the inhibition of fungal spore germination and destruction of the cell membrane that caused leakage of intracellular proteins and nucleic acids. Therefore, the limonin-loaded eugenol nanoemulsion, with effective antifungal activity against *P. italicum*, could be considered as an alternative fungicide to inhibit blue mold in citrus fruits.

## Figures and Tables

**Table 1:** MIC and MFC of EG and EGL nanoemulsions against *P. italicum.*

Groups	Concentrations ^a^	Mycelial Growth in PDB (3rd Day)	Mycelial Growth on PDA (6th Day)	MIC ^a^	MFC ^a^
EG	1280	−	−	320	320
	640	−	−		
	320	−	−		
	160	+			
	80	+			
EGL	1280	−	−	160	320
	640	−	−		
	320	−	−		
	160	−	+		
	80	+			
PDB + Stain	0	+			
PDB	0	−			

“+” refers to the presence of mycelial growth, whereas “−” refers to the absence of mycelial growth. ^a^ Concentration is expressed as μg/mL.

**Table 2:** Percentages *^n^* (%) of the infected oranges and infected cuts inoculated with *P.italicum.*

Groups	Infected Oranges (%)		Infected Cuts (%)	
	3 d	5 d	3 d	5 d
CK	75.0 ± 12.5 ^a^	100.0 ± 0.0 ^a^	59.7 ± 6.4 ^a^	84.7 ± 6.4 ^a^
EG	37.5 ± 12.5 ^b^	58.3 ± 7.2 ^b^	29.2 ± 11.0 ^b^	38.9 ± 8.7 ^b^
EGL	20.8 ± 7.2 ^b^	29.2 ± 7.2 ^c^	12.5 ± 4.2 ^c^	19.4 ± 6.4 ^c^

*^n^* The percentages are expressed as mean ± SD for three replicates. Different letters in the same column indicate a significant difference at *p* < 0.05.

## Data Availability

The data presented in this study are available on request from the corresponding author.

## Supplementary Materials

The following are available online at https://www.mdpi.com/article/10.3390/microorganisms9050969/s1, Figure S1: Colony morphology and mycelia status of *P. italicum* grown in PDA plate (5 d) and in PDB medium (3 d) under different treatments, Figure S2: A diagram to show how the citrus fruits were cut and injected in the challenge test.
