# Supplementary material for: Limonin Enhances the Antifungal Activity of Eugenol Nanoemulsion against Penicillium Italicum In Vitro and In Vivo Tests
Source: Microorganisms. 2021 Apr 30;9(5):969. doi: 10.3390/microorganisms9050969 (PMC8144956; doi:10.3390/microorganisms9050969)
Supplement: Supplementary file 1 [file microorganisms-09-00969-s001.zip › microorganisms-1179813-supplementary.pdf]

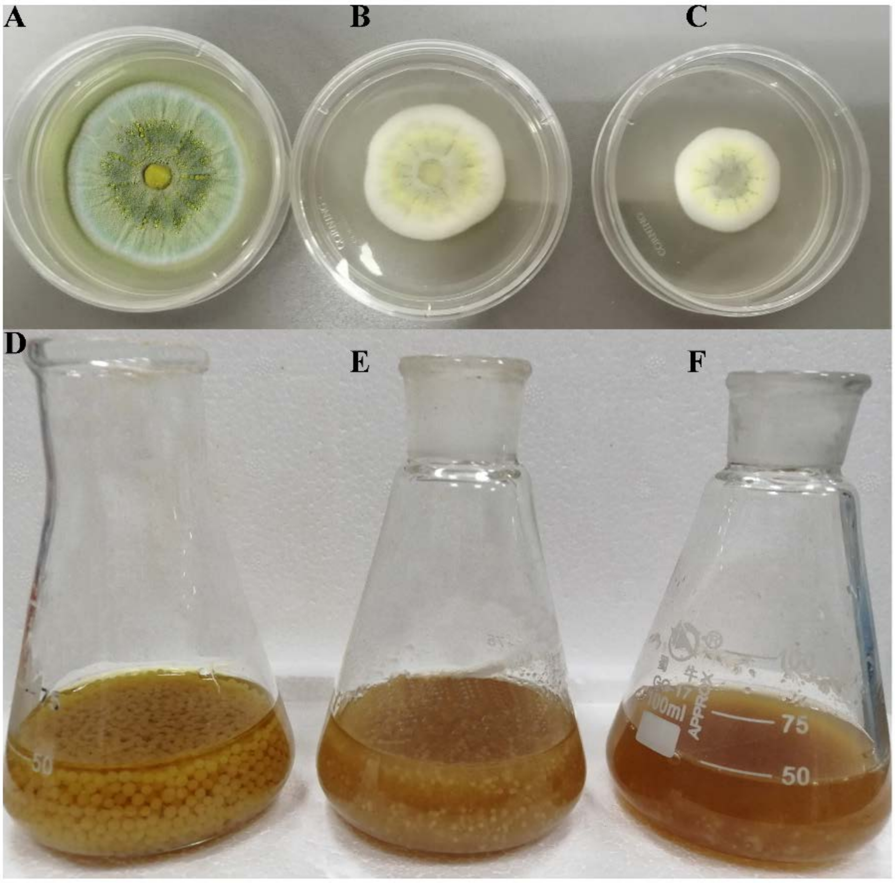

Figure S1. Colony morphology and mycelia status of P. italicum grown in PDA plate (5 d) and in PDB medium (3 d) under different treatments. **A**,**D** were treated with sterile water (CK), **B**,**E** were treated with 160 μg/mL EG nanomulsion (EG), **C**,**F** were teated with 160 μg/mL EGL nanoemulsion(EGL).

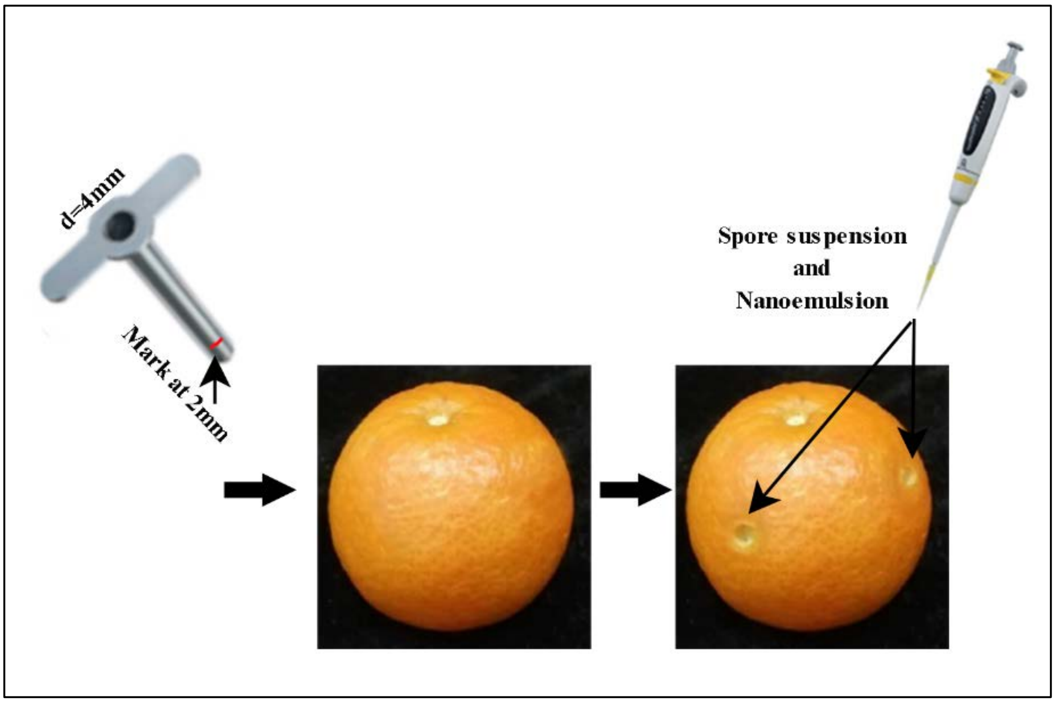

Figure S2. A diagram to show how the citrus fruits were cut and injected in the challenge test. A sterile punch (d = 4mm) was used to cut holes in the fruits, and spore suspension and nanoemulsions were added into the holes by a micropipette.
